# Non-prescription sale and dispensing of antibiotics for prophylaxis in broiler chickens in Lusaka District, Zambia: findings and implications on one health

**DOI:** 10.1093/jacamr/dlae094

**Published:** 2024-06-11

**Authors:** Steward Mudenda, Karen Mubanga Mulenga, Ruth Nyirongo, Billy Chabalenge, Chikwanda Chileshe, Victor Daka, Ethel M’kandawire, Elimas Jere, John Bwalya Muma

**Affiliations:** Department of Pharmacy, School of Health Sciences, University of Zambia, Lusaka, Zambia; Department of Pharmacy, School of Health Sciences, University of Zambia, Lusaka, Zambia; Department of Pharmacy, School of Health Sciences, University of Zambia, Lusaka, Zambia; Department of Medicines Control, Zambia Medicines Regulatory Authority, Lusaka, Zambia; Department of Biomedical Sciences, School of Veterinary Medicine, University of Zambia, Lusaka, Zambia; Department of Disease Control, School of Veterinary Medicine, University of Zambia, Lusaka, Zambia; Department of Public Health, Michael Chilufya Sata School of Medicine, Copperbelt University, Ndola, Zambia; Department of Post Marketing Surveillance, Zambia Medicines Regulatory Authority, Lusaka, Zambia; Department of Public Health, Michael Chilufya Sata School of Medicine, Copperbelt University, Ndola, Zambia

## Abstract

**Background:**

The irrational use of antibiotics in humans and livestock has contributed to the emergence of antimicrobial resistance (AMR). This study investigated the commonly sold and dispensed antibiotics for prophylaxis in broiler chickens in pharmacy and agro-veterinary shop personnel in the Lusaka District of Zambia.

**Methods:**

This cross-sectional study was conducted from August 2023 to October 2023 among 200 veterinary medicine dispensers in the Lusaka District of Zambia using a simulated farmer or mystery shopper approach. Data analysis was performed using IBM Statistical Package for Social Sciences version 23.0.

**Results:**

Out of the 200 medicine outlets investigated, 23 (11.5%) were agro-veterinary shops, while 177 (88.5%) were community pharmacies. A total of 165 community pharmacies and agro-veterinary shops provided veterinary services in the Lusaka District and sold medicines without prescription giving a 100% non-prescription sale. Of the 178 medicines dispensed for prophylaxis, 88.5% were antibiotics, while 13.5% were vitamins. The most dispensed antibiotic drug for prophylaxis in broiler chickens was oxytetracycline (30.34%), amoxicillin (17.98%) and gentamicin/doxycycline (10.67%).

**Conclusions:**

This study revealed a high sale and dispensing of antibiotics for prophylaxis in broiler chickens of which oxytetracycline, amoxicillin and gentamicin/doxycycline were commonly dispensed. There is a need for increased regulatory enforcement of selling antibiotics without a prescription as this may predispose poultry to the development of AMR and possible transmission of superbugs to humans and the environment. Educational interventions should be provided to community pharmacy and agro-veterinary personnel on adhering to antimicrobial stewardship practices when dispensing poultry antibiotics.

## Introduction

Antibiotics are frequently used in the poultry sector for growth promotion, prophylaxis and treatment of infections.^1^ A study reported that global consumption of antibiotics in food-producing animals will increase by 67% by the year 2030.^2^ Consequently, this may lead to the emergence and spread of antibiotic-resistant pathogens in the animal sector with potential adverse impacts on humans.^1,2^ This may contribute to a rise in antimicrobial resistance (AMR).^1,3^

Poultry production is greatly expanding to meet the rising demand for meat and eggs for human consumption.^4^ In poultry, the chicken industry includes the rearing and selling of broilers and layers to generate income and for nutrition purposes.^1,4^ However, due to this increase in demand for poultry products such as eggs and chicken meat, farmers tend to use antibiotics for prophylaxis and therapeutic purposes.^3–5^ In sub-Saharan Africa (SSA), the purchasing of antibiotics without a prescription is a huge problem.^6–8^ Consequently, a high prevalence of drug-resistant pathogens has been reported in the poultry sector across the SSA region.^3^ Unfortunately, the overuse and misuse of antibiotics in the poultry sector are likely to worsen the AMR problem in the SSA region.^3^

In Zambia, the Medicines and Allied Substances Act No. 3 of 2013 prohibits the supply or sale of prescription-only medicines (PoM) without a prescription.^9^ However, inadequate enforcement of this provision by relevant bodies, poor dispensing attributes of healthcare professionals and health self-seeking behaviours among the public have made it very easy to access antibiotics without a prescription.^8,10^ Previous studies done in Zambia have reported that antibiotics for poultry use are usually accessed without prescriptions.^10–12^ However, there is little information on the use of antibiotics for prophylaxis in broiler chickens in Zambia.

Therefore, this study investigated the commonly sold and dispensed antibiotics for prophylaxis in broiler chickens in community pharmacies and agro-veterinary shops in Lusaka District, Zambia.

## Materials and methods

### Study design, settings and population

This cross-sectional study was conducted in community pharmacies and agro-veterinary shops in the Lusaka District of Zambia from August 2023 to October 2023 using a mystery shopping approach. Additionally, only business owners and participants who agreed to participate and provided written and verbal consent before conducting the study were included in the study. The study sites were registered by the Zambia Medicines Regulatory Authority (ZAMRA) in the Lusaka District, Zambia.^13^ In Zambia, all antibiotics are classified as PoM and should only be sold and dispensed on prescription.^9^

### Sample size estimation

The sample size was determined using Taro Yamane’s formula. The study population was based on the 370 community pharmacies and 30 agro-veterinary shops that were registered by the ZAMRA in the Lusaka District, Zambia.^13^

### Data collection

The data were collected using a tool that was adopted from a simulated client study.^14^ In the current study, we used two simulated farmers (SFs) to purchase prophylactic medicines for poultry use in community pharmacies and agro-veterinary shops.

### Data analysis

The collected data were entered into Microsoft Excel 2013 and later exported to Statistical Package for Social Sciences version 23.0 for analysis.

### Ethical approval

Ethical approval was obtained from the University of Zambia Health Sciences Research Ethics Committee through the Department of Pharmacy with a protocol approval number of 202301270012.

## Results

**Figure 1.**
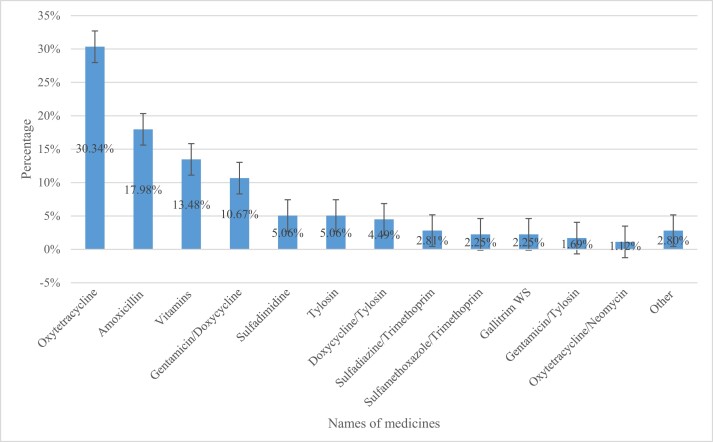
Names of medicines dispensed for prophylaxis in broiler chickens. Gallitrim, erythromycin/sulfadiazine/trimethoprim; Aliseryl, erythromycin/streptomycin/oxytetracycline/colistin/vitamins; Other, Aliseryl WS, amoxicillin/colistin, amprolium, fosfomycin and neomycin.

Out of the 200 sites investigated, 23 (11.5%) were agro-veterinary shops and 177 (88.5%) were community pharmacies. Overall, 165 of the 200 sites stocked veterinary medicines, and all 165 of these sites sold antibiotics without a prescription. Of the 178 medicines that were sold, 86.5% were antibiotics. The commonly dispensed antibiotics were oxytetracycline (30.34%), amoxicillin (17.98%) and gentamicin/doxycycline (10.67%) (Figure 1).

**Figure 2.**
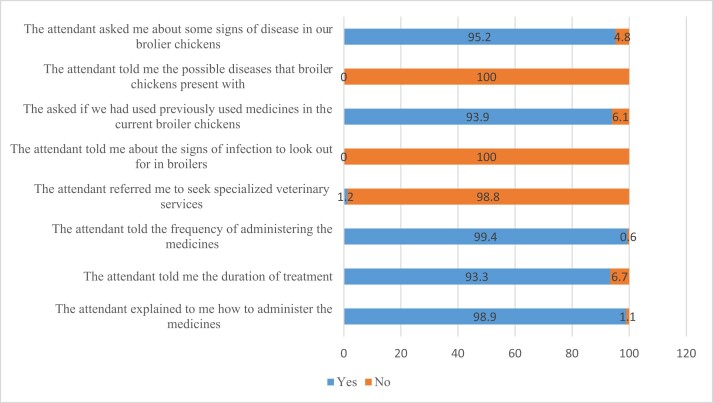
Counselling the farmer concerning diseases in broilers and administration of medicines to sick birds.

Out of the 165 attendants in community pharmacies and agro-veterinary shops that stocked veterinary medicines, 95.2% asked the SFs whether the broiler chickens presented with any signs of disease (Figure 2). Additionally, 93.9% of the attendants asked the SFs about previous medicines used in the broiler chickens. None (0%) of the attendants told the SFs the names of diseases found in broilers, whereas 98.8% of attendants did not refer the SF for specialized services to the veterinarians (Figure 2). Most of the attendants counselled SFs concerning the frequency and duration of treatment and how to administer medicines to sick birds (Figure 2).

## Discussion

This study investigated the commonly dispensed antibiotics for prophylaxis in broilers in community pharmacies and agro-veterinary shops in the Lusaka District of Zambia. The prevalence of antibiotic sale and dispensing was 86.5%. All (100%) of the antibiotics were sold and dispensed antibiotics without a prescription. Similar findings have been reported in research conducted in Ghana, Tanzania and Kenya, where drug sellers dispensed most antibiotics used in poultry without a prescription.^3,5,7^ This may be attributed to inadequate enforcement of regulations resulting in easy access to antibiotics, where people easily buy antibiotics without a prescription, as reported in a study that was conducted in Ghana.^5^ The non-prescription sale of antibiotics has also been reported in the human health sector, where there were a 100% non-prescription sale of antibiotics in community pharmacies in Zambia^8^ and 76.5% in Benin,^15^ respectively. The non-prescription sale of medicines is a global problem with negative impacts on animals, humans and the environment.^16,17^ In poultry production, the increased use and misuse of antibiotics is a worldwide problem due to an increase in the global demand for chicken meat and also to promote the growth and well-being of broiler chickens.^4,5,7,11,18^ Consequently, this increased use and misuse may lead to the development and spread of AMR in poultry.^1^

Our study revealed that 86.5% of the sold and dispensed medicines were antibiotics. This is in contrast to a study that was conducted in Bangladesh where vitamins were the most prominently dispensed (72%), followed by antibiotics (41%) and probiotics (30%).^4^ The widespread use of antibiotics can be attributed to the ease of access and purchase from drug stores with or without a prescription.^5,6^ Previous studies in Zambia have shown that the prevalent use of antibiotics in the poultry sector has led to the report of AMR strains of microorganisms in the poultry sector, which can then be transmitted to humans via the environment, food products and direct contact with food-producing animals.^11,19^

The present study found that most of the dispensed antibiotics for prophylactic use in broilers included tetracyclines, penicillins, aminoglycosides, sulphonamides, polymyxins and macrolides. This is consistent with a recent study that was conducted in Zambia, where tetracyclines, aminoglycosides, penicillins and sulphonamides were highly used in poultry.^12^ Our study found that the most commonly dispensed antibiotics were oxytetracycline, amoxicillin and gentamicin/doxycycline, which is similar to previous studies conducted in Zambia,^12^ Malawi^20^ and Bangladesh,^4^ respectively. Our findings contrast with what was found in Kenya, where aliseryl was the most dispensed drug for prophylactic use.^7^ These findings indicate the high use of antibiotics that are also commonly used in humans.

Our study found that despite most of the attendants in the community pharmacies and agro-veterinary shops asking the SF about signs of disease noticed in broilers and counselling on medicine use in sick birds, very few of them referred the clients to veterinary experts for specialized services. An earlier study that was conducted in Zambia found that most pharmacy professionals referred farmers to animal health workers for specialized services.^12^ This indicated a good collaboration between pharmacy professionals and veterinarians, which could help reduce the inappropriate use of antibiotics in poultry.^12^ Referring farmers to veterinary personnel is essential because they understand animal diseases in detail.

We believe the access to poultry medicines translates to the importance of community pharmacies and agro-veterinary shops in providing a service to poultry farmers. This is significant because it helps poultry farmers to access the medicines needed to prevent and treat infections in their flock. Consequently, there is a need to deregulate some antibiotics so that farmers can have access without challenges. Subsequently, there is a need to provide educational activities to community pharmacy professionals and veterinary professionals to promote rational dispensing of poultry antibiotics. Finally, we recommend the development and implementation of antibiotic prescribing and treatment guidelines in the poultry sector in Zambia.

We are aware of the limitations of this study. Since this study was conducted in one district across the country, the findings may not be generalized to the rest of the districts in the country. However, the findings are critical to policymakers, regulators, professional bodies and farmers to the need to restrict the use of antibiotics for prophylaxis in chickens. Despite antibiotics being very important, there is no evidence of their effectiveness for prophylaxis in the poultry sector. Hence, there is a need to limit the sale of poultry antibiotics to the general public as PoM, as this will help reduce the potential emergence of drug-resistant infections. To achieve this, relevant government agencies must work with professional bodies to effectively enforce provisions that prohibit the supply of PoM without a prescription from authorized healthcare professionals.

### Conclusions

This study found that antibiotics were the most commonly sold and dispensed medicines in community pharmacies and agro-veterinary shops for prophylactic use in broiler chickens. The study revealed a high sale of antibiotics without a prescription with oxytetracycline being the most sold and dispensed antibiotic. There is a need to instigate and implement antimicrobial stewardship programmes in community pharmacies and agro-veterinary shops to promote rational dispensing of medicines used in poultry and other animal health sectors. Additionally, there is a need to heighten the regulations that restrict the overuse and misuse of antibiotics in poultry.

## Transparency declarations

The authors have no relevant conflicts of interest to declare. All the authors do not have any financial interests or connections that may directly or indirectly raise concerns of bias in the work reported or the conclusions, implications or opinions made in this publication. The authors declared no potential conflicts of interest concerning the research, authorship, and/or publication of this article. The authors declare that the research was conducted in the absence of any commercial or financial relationships that could be construed as a potential conflict of interest.
